# Testosterone Increases Susceptibility to Amebic Liver Abscess in Mice and Mediates Inhibition of IFNγ Secretion in Natural Killer T Cells

**DOI:** 10.1371/journal.pone.0055694

**Published:** 2013-02-12

**Authors:** Hannelore Lotter, Elena Helk, Hannah Bernin, Thomas Jacobs, Cornelia Prehn, Jerzy Adamski, Nestor González-Roldán, Otto Holst, Egbert Tannich

**Affiliations:** 1 Bernhard Nocht Institute for Tropical Medicine, Hamburg, Germany; 2 Helmholtz Center Munich, Institute of Experimental Genetics, Genome Analysis Center, Neuherberg, Germany; 3 Division of Immunobiology, Research Center Borstel, Leibniz-Center for Medicine and Biosciences, Borstel, Germany; 4 Division of Structural Biochemistry, Research Center Borstel, Leibniz-Center for Medicine and Biosciences, Borstel, Germany; Institut national de la santé et de la recherche médicale - Institut Cochin, France

## Abstract

Amebic liver abscess (ALA), a parasitic disease due to infection with the protozoan *Entamoeba histolytica*, occurs age and gender dependent with strong preferences for adult males. Using a mouse model for ALA with a similar male bias for the disease, we have investigated the role of female and male sexual hormones and provide evidence for a strong contribution of testosterone. Removal of testosterone by orchiectomy significantly reduced sizes of abscesses in male mice, while substitution of testosterone increased development of ALA in female mice. Activation of natural killer T (NKT) cells, which are known to be important for the control of ALA, is influenced by testosterone. Specifically activated NKT cells isolated from female mice produce more IFN**γ** compared to NKT cells derived from male mice. This high level production of IFN**γ** in female derived NKT cells was inhibited by testosterone substitution, while the IFN**γ** production in male derived NKT cells was increased by orchiectomy. Gender dependent differences were not a result of differences in the total number of NKT cells, but a result of a higher activation potential for the CD4^−^ NKT cell subpopulation in female mice. Taken together, we conclude that the hormone status of the host, in particular the testosterone level, determines susceptibility to ALA at least in a mouse model of the disease.

## Introduction

Susceptibility to and outcome of infectious diseases may be greatly influenced by the gender. There are various examples, in particular for parasitic diseases, in which male individuals are more frequently infected with the respective pathogen, suffer from higher parasite burden or develop more severe clinical courses in comparison to females [1], [2]. An extraordinary example for a male bias towards a parasitic disease is amebic liver abscess (ALA). The disease is endemic in most tropical and subtropical countries and is characterized by massive liver tissue destruction due to infection with the protozoan *Entamoeba histolytica*. This parasite primarily colonizes the human gut where it can reside and multiply for months or even years without inducing any clinical symptoms. However, in a small proportion of infected individuals the parasite invades the tissue and causes colitis or extraintestinal abscesses, most commonly in the liver. Interestingly, ALA is rare in children and women. The vast majority of more than 80% of all ALA cases occur in adult males [3], [4], [5]. This phenomenon is independent from ethnic or cultural background as it is found in all parts of the world in which ALA is endemic as well as in travelers from non-endemic countries acquiring the disease during their journey [6], [7], [8]. In one of the largest studies on the epidemiology of ALA in Central Vietnam, in which more than 2,000 ALA cases were analyzed, it was shown that the risk for ALA increases after puberty with peak incidence in adult males at the age between 30 and 50 years, suggesting that sexual hormones in particular testosterone might have an impact on ALA susceptibility [4], [5], [9].

The influence of steroid hormones on the outcome of parasitic diseases, either by direct interactions with the parasite or by altering immune functions is well documented [2]. In animal models for *leishmaniasis or malaria*, testosterone treatment decreases the resistance to parasite infections [10], [11] either by suppressing functions of innate immune cells [12] or by promoting anti-inflammatory immune responses [13], [14], [15], [16]. Interestingly, resistance to ALA mainly relies on a pro inflammatory type of immune response primarily based on the production of IFN**γ**. The importance of IFN**γ** in the early control of *E. histolytica* invasion has been documented from various *in vitro* studies as well as from animal models for experimental ALA [17], [18], [19], [20].

One of the main producer of IFN**γ** in early response to microbial infections are natural killer T (NKT) cells, which are immune cells, bridging the early innate and the adaptive immune response. NKT cells are activated by a range of microbial-derived lipids and glycolipids that are presented by the major histocompatibility complex (MHC) class I-like molecule CD1d on antigen presenting cells (APCs) [21], [22]. Upon activation, NKT cells exhibit regulatory functions by rapidly producing pro- and anti- inflammatory cytokines and mediate direct cytotoxicity against microbial pathogens [23], [24], [25], [26]. Recently, we have shown that murine NKT cells could be activated to produce protective IFN**γ** by an *E. histolytica* lipopetidophosphoglycan (EhLPPG) present on the surface of ameba trophozoites [27]. In a C57BL/6 mouse model for ALA, knockout mice lacking functional NKT cells are impaired in their ability to control ALA [20], whereas activation of NKT cells by EhLPPG or by their most potent activator α-galactosylceramide (αGalCer) limit ALA development [27]. Interestingly, the C57BL/6 ALA mouse model revealed a strong sexual dimorphism in the susceptibility for ALA, mimicking at least in part the human situation. In this mouse model, female mice are able to clear parasites injected into the liver within 3 days, whereas in male mice viable ameba can be isolated up to 14 days. Accordingly, male mice develop larger abscesses and show a prolonged recovery from ALA [20].

In this communication, we report on the use of the C57BL/6 ALA mouse model to analyze the role of sexual hormones for ALA susceptibility and provide evidence that testosterone increases susceptibility for ALA by modulation the secretion of IFN**γ** by EhLPPG-activated NKT cells.

## Materials and Methods

### Ethic Statement

Mice were maintained in a specific pathogen-free microenvironment in the animal facility of the Bernhard-Nocht-Institute and received care in compliance with the European Union guidelines for the handling of laboratory animals (http://ec.europa.eu/environment/chemicals/lab_animals/home_en.htm). All work was conducted with the approval of the Behörde für Gesundheit und Verbraucherschutz der Stadt Hamburg according to §8 TierSchG (German Protection of Animals Act; # 20/08; # 23/09; # 23/11).

### Cultivation of Parasites and Preparation of EhLPPG

Trophozoites of the *E. histolytica* isolate HM1:IMSS were axenically grown in TY-S-33 medium (Diamond 1978). Parasites from the log growth stage were used for intrahepatic infection of C57BL/6 mice.

EhLPPG was purified from the membranes of *E. histolytica* trophozoites as described previously [27]. In brief, trophozoites of the late logarithmic phase of growth were washed, resuspended in pyrogen-free water and lysed by freeze and thawing. The homogenate was centrifuged at 430 *g* at 4°C for 10 min and subsequently the supernatant was recovered and the trophozoite membranes were enriched by ultracentrifugation at 150,000 *g* for 40 min. The obtained pellet was extracted with a mixture of chloroform/methanol/water 10∶10:3 (by volume) and the insoluble material was recovered by centrifugation, dried, resuspended in distilled pyrogen free water and extracted three times with an equal volume of 90% phenol at 68°C for 30 min with constant stirring [28]. The water phase containing EhLPPG was recovered after centrifugation at 12,000 *g* for 30 min and dialysis against distilled water.

### Ovariectomy, Orchiectomy and Hormone Treatment

Female and male C57BL/6J mice at an age of 8 weeks were used for the experiments. The following groups of mice were assembled:

Naive mice, ovariectomized (ovx) mice supplemented with testosterone, ovariectomized and orchiectomized mice treated with placebo, non-ovariectomized mice supplemented with testosterone and non-ovariectomized mice treated with placebo.

Female mice were ovariectomized at an age of 8 weeks. The mice were prepared for surgery using an anesthetic protocol that enabled the abrogation of the narcosis immediately after finishing the surgical intervention. Inititially, a solution consisting of 6.3 µl Domitor (Pfizer, Karlsruhe, Germany,), 27 µl Ketanest (Inresa, Freiburg, Germany) and 66 µl phosphate buffered saline (PBS) was injected intraperitoneally. The tolerance time using this method was approximately 60 min. The intra muscular injection of 5 µl Antisedan (Pfizer)/45 µl phosphate buffered saline/animal abrogated the narcosis within the next 10 min.

After skin desinfection, a dorsal midline skin incision was made caudal to the border of the ribs. Lateral to the incision, the muscle layer of the posterior abdominal wall and the peritoneum were separated to enter the abdominal cavity. The periovarian fat tissue was grasped of to lift and exteriorize the ovary. The cranial fixation of the ovary and the arteria ovarica, as well as the fallopian tube were carefully heat coagulated and the ovary was removed. The blunt end of the uterine horn was slowly relocated in the abdominal cavern. When no further bleedings were observed, the peritoneum and the muscle layers were sutured and the skin was closed using skin clamps. The process was then repeated to remove the second ovary.

Surgical orchiectomy was performed via a midline scrotal incision allowing bilateral access to the hemiscrotal contents. After exposing each testicle, a 3-0 vicryl suture was used to ligate the spermatic cord and then remove the testicle.

For hormone treatment, female mice were implanted subcutaneously with pellets releasing testosterone designed to yield blood levels of 6–9 ng/ml (12.5 mg/pellet/60 day release, Innovative Research of America, Sarasota, FL, USA). Control mice were implanted with Placebo pellets for testosterone (12.5 mg/pellet/60 day release, Innovative Research of America, Sarasota, FL, USA).

### Induction of Amebic Liver Abscess

The mice were infected by direct hepatic inoculation of virulent trophozoites as previously reported [20]. Following anesthesia and desinfection of the surgery area, the skin, the muscle and the peritoneal layer were incisized to enter the abdominal cavern. 1×10^5^ axenically cultured trophozoites in a volume of 25 µl TY medium were injected in the visualized left liver lobe using a U-100 insulin syringe (BD, Heidelberg, Germany). Peritoneum and muscle layer were sutured and wound clips closed the skin. To maintain virulence, trophozoites were regularly passaged through the liver of male C57BL/6 mice. On day 7 post infection, the mice were sacrificed, the abscess size was calculated by measuring the size in mm and the introduction of score values (score 0: no abscess; score 1: abscess <1 mm; score 2: >1–<5 mm; score 3: >5 mm) and the abscess material was maintained in TY medium at 37°C to provide optimal conditions for the re-isolation of amebic trophozoites.

### Determination of Testosterone Concentrations in Serum Samples

Since no steroid ELISA kits are available for mouse samples, a human ELISA kit has been adopted. Prior to the measurement, steroids were extracted from the matrix by liquid/liquid-extraction to avoid mouse plasma matrix effects. The plasma was extracted three times in each case with a tenfold excess of tetra-butylmethylether (TBME). After evaporation of the combined organic extracts the dried residues can be stored at −20°C. For the subsequent ELISA, the material is reconstituted in steroid free serum. The serum testosterone concentration was quantified using a competitive ELISA according to the manufacturer’s protocols (testosterone EIA-1559; DRG Instruments GmbH, Germany). The plates were read in a standard microplate reader at 450 nm (Tecan, Safire 2). The concentrations were calculated upon the calibration from the standard curve and reported in ng/ml. The sensitivity of the testosterone assay is 0.083 ng/ml.

### Isolation of Murine Liver Lymphocytes and FACS Sorting of NKT Cells

Livers were perfused with ice cold PBS/20% FCS solution and subsequently filtered through a 40 µm mesh. Following centrifugation at 400 *g*, cell pellets were resuspended in RPMI medium, underlayed with a 30% Nycodenz solution (Nycoprep, Universal) and centrifuged at 900 *g* for 20 min. The liver lymphocytes were collected from the interface, treated with hypotonic ammonium chloride solution, washed and diluted in RPMI medium.

Gradient purified liver lymphocytes were further characterized by staining with APC-labelled anti-CD3, PE-labelled CD11b and FITC-labelled anti-GR1 in phosphate buffered saline containing 1% bovine serum albumine. The cells were subsequently subjected to flow cytometry on a FACS Calibur (BD Bioscience, Heidelberg, Germany) and the data were analyzed using the CellQuestPro software (BD Bioscience).

NKT-cells were stained for cell sorting using αGalCer (Alexis Biochemicals, Lausen, CH) loaded to PE labelled recombinant CD1d-tetramer (Proimmune, Oxford, UK) and FITC- labelled anti-CD4 (BD Bioscience, Heidelberg, Germany). Cell sorting was performed on a FACS Aria III (BD).

### *In vitro* Stimulation of Murine NKT Cells

Generation of APCs. Bone marrow was harvested from femurs of male and female, 6- to 10-weeks-old C57BL/6 mice and cultured as described by Lutz et al. [29]. Cultures were supplemented with supernatants from Ag8653 myeloma cells transfected with the gene coding for murine GM-CSF [30]. The maturity of the bone marrow derived dendritic cells (BMDCs) was determined by FACS analysis using APC- labelled anti- CD11c, FITC- labelled anti - CD40-FITC and PE- labelled anti - CD86 and anti - CD80 (BD Bioscience). The ratio of immature to mature BMDCs was 80% vs 20%.

*In vitro* stimulation of liver lymphocytes from testosterone treated female mice and orchiectomized male mice. In brief, gradient purified liver lymphocytes from hormone-modified mice were subjected to the PAN T cell isolation kit (MACS, Miltenyi Biotec, Bergisch Gladbach, Germany) according to the manufacturers instructions. Purified T cells (1×10^5^) were added to gender matched APCs (5×10^4^) previously pulsed with 4 µg/ml αGalCer and 8 µg/ml EhLPPG for 3 h. The cells were cultured in triplicates in 96-well round bottom plates with RPMI-20 medium supplemented with 200 mM L-glutamine, 50 µg gentamicin, 10% fetal calf serum, 1 mM sodium pyruvate and, 50 µM 2-mercaptoethanol. Cell supernatants were collected after 48 h of coincubation and assayed for IFN**γ** by enzyme-linked immunosorbent assay (ELISA) using antibody pairs purchased from R&D Systems (Abington, UK).

*In vitro* stimulation of purified murine NKT cells. Gradient purified liver lymphocytes from female and male C57BL/6 mice (10–12 weeks old) were stained with PE-labelled- αGalCer -CD1d-tetramer, sorted by fluorescence activated cell sorting (BD-FACS-Aria) and added in a cell density of 5×10^4^ to 1×10^4^ gender matched APCs prepulsed for 3 h with either αGalCer (4 µg/ml) or purified amebic EhLPPG (8 µg/ml). The cell supernatant was assayed for IFN**γ** as described above.

*In vitro* stimulation assay using *i*NKT cell subpopulations. Gradient purified liver lymphocytes from female and male C57BL/6 mice (10–12 weeks old) were stained with PE-labelled- αGalCer -CD1d tetramer (Proimmune) and FITC-labelled anti-CD4. Tetramer positive, CD4^+^ or tetramer positive, CD4^−^ NKT cells were sorted by fluorescence activated cell sorting (BD-FACS-Aria) and added at a cell density of 3×10^4^ to 1×10^4^ gender matched APCs previously pulsed with αGalCer or EhLPPG as described above. The amount of IFN**γ** was measured by ELISA in the cell supernatant after 48 h of co-cultivation.

### Statistical Analysis

The Mann Whitney U test was applied to compare the liver abscess sizes between male and female mice over the time period monitored and the Paired t-test was used to determine the difference between abscesses that were culture positive for *E. histolytica*. The student`s t test was applied for the statistical analysis of the IFNγ values obtained by the NKT cell activation assay.

## Results

### Gonadectomy Decreases Susceptibility for ALA in Male Mice

**Figure 1:**
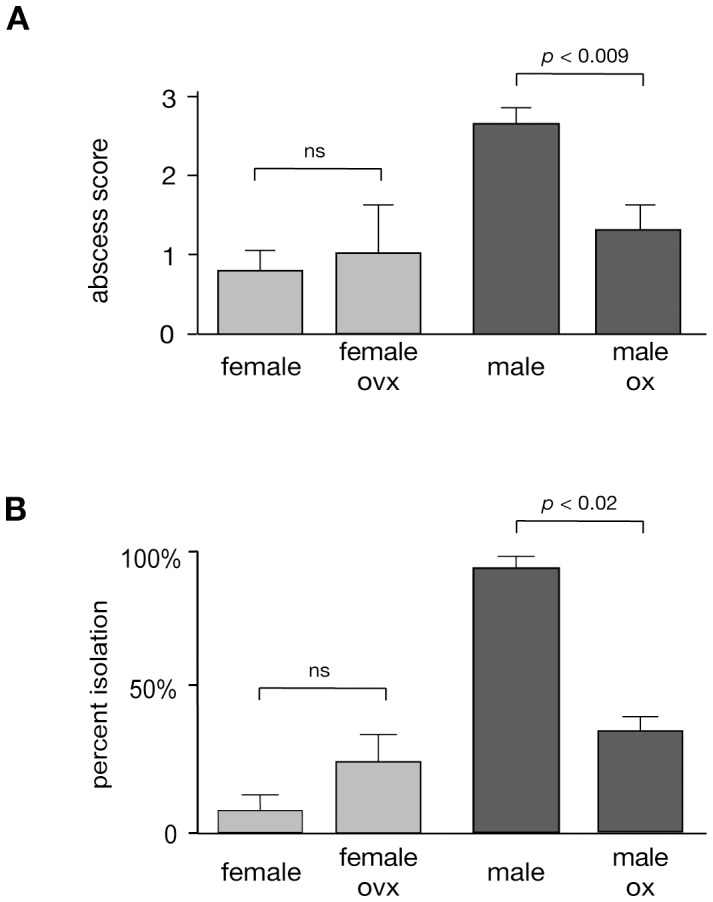
Influence of gonadectomy on the size of ALA in the mouse model for the disease. Eight weeks old male and female mice were gonadectomized and intrahepatically challenged with 1×10^5^ *E. histolytica* trophozoites seven weeks after castration. The animals were sacrificed 7 days post infection, the sizes of abscesses were determined and transformed into score values (score 0: no abscess; score 1: abscess <1 mm; score 2∶1 to 5 mm; score 3: >5 mm) (Fig. 1A) or the re-isolation rate of live *E. histolytica* trophozoites from abscessed liver tissue was determined (Fig. 1B). Results were obtained from 3 independent trials each comprising 7 animals (statistics: Mann Whitney U test).

The C57BL/6 mouse model for ALA is characterized by the development of significant larger abscesses in male mice within 7 days following intraheptic ameba challenge, whereas female mice control the infection and develop only small lesions. To investigate whether sexual hormones might have an influence on ALA development, gonadectomy was performed in groups of 8 weeks old male and female mice. Seven weeks following castration, animals were challenged by intrahepatic application of cultured *E. histolytica* trophozoites. Development of ALA was assessed on day 7 after infection (Fig. 1). The results clearly indicate significant reductions in the sizes of ALA (*p*<0.009) and parasite survival rates (*p*<0.02) in orchiectomized male mice compared to respective male controls. The sizes of ALA in orchiectomized mice were similar to those found in female mice. In contrast, ovariectomy did not influence sizes of ALA or parasite survival rates in female mice suggesting that testosterone rather than estrogens influences susceptibility to ALA.

### Substitution of Testosterone Increases Susceptibility for ALA in Female Mice

**Figure 2:**
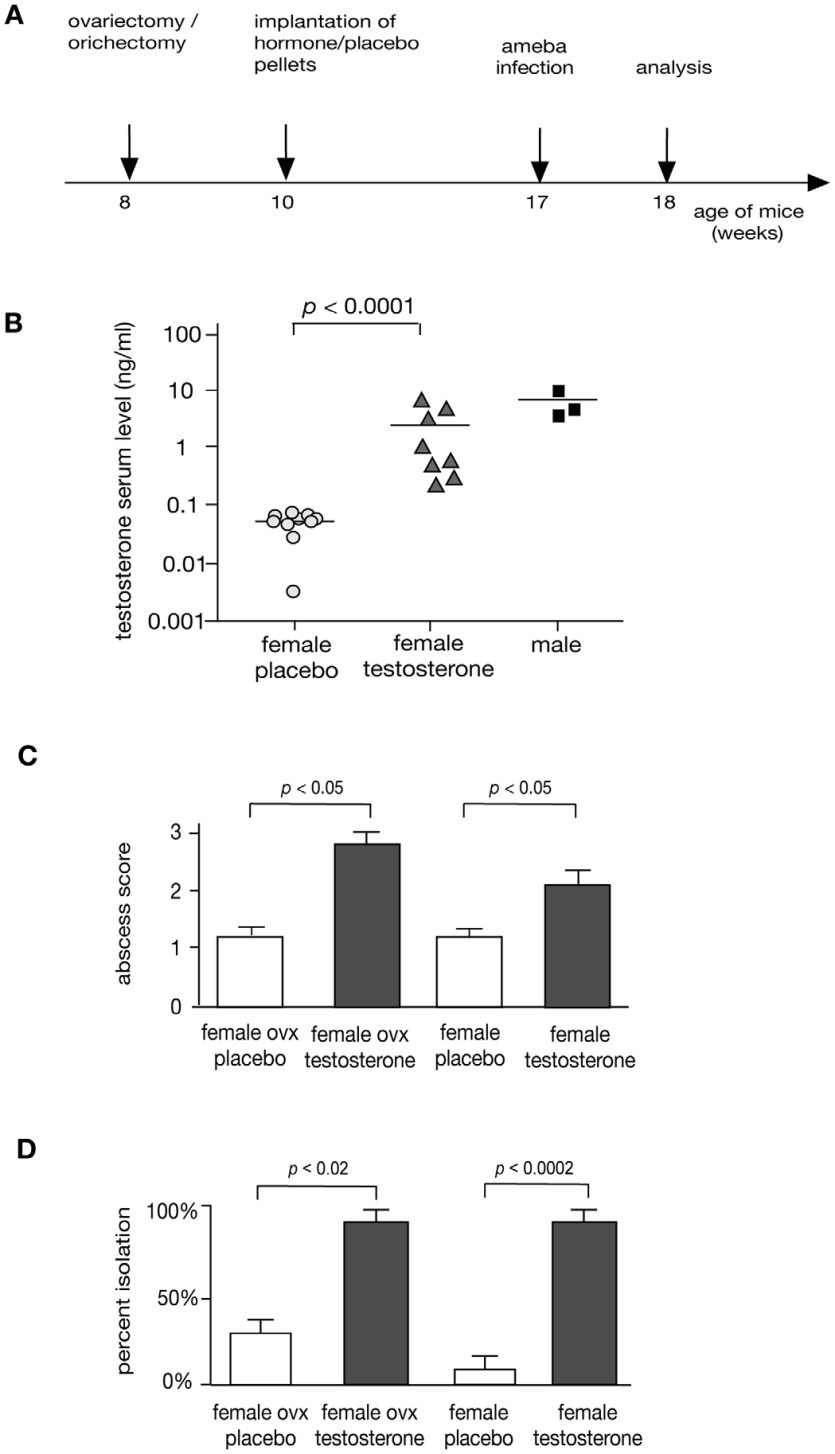
Influence of testosterone on the development of ALA. A) Time schedule of gonadectomy, testosterone substitution and intrahepatic infection with *E. histolytica* trophozoites. B) Serum testosterone levels in placebo treated naïve female mice, testosterone substituted female mice and male mice measured by ELISA (ng/ml). C) Size of ALA in testosterone treated, ovariectomized (ovx) or naïve female mice. Score values indicative for the size of ALA determined on day 7 post intrahepatic infection with 1×10^5^ virulent *E. histolytica* trophozoites of naïve female mice treated either with testosterone or placebo are shown (score 0: no abscess; score 1: abscess <1 mm; score 2∶1 to 5 mm; score 3: >5 mm). D) Re-isolation rate of *E. histolytica* trophozoites from abscessed liver tissue of ovariectomized (ovx) and naïve female mice treated either with testosterone or placebo. Results were obtained from at least 3 independent trials each comprising of 7 animals (statistics: Mann Whitney U test).

To further determine the importance of testosterone for ALA development, testosterone substituted female mice were investigated. Substitution was performed by subcutaneous implantation of small pellets releasing either testosterone or placebo. Pellets were implanted into 10 weeks old C57BL/6 female mice or into female mice following ovariectomy (Fig. 2A). Seven weeks later, prior to intrahepatic ameba challenge, serum testosterone concentrations were determined indicating similar levels between male and testosterone-substituted female mice (Fig. 2B). Intrahepatic infection of the various groups of testosterone and placebo substituted animals revealed significant increases of abscess sizes in testosterone-substituted animals compared to sham-substituted controls (*p*<0.05), regardless whether mice were ovariectomized or not (Fig. 2C). Moreover, 90% of abscesses of testosterone treated mice contained viable *E. histolytica* trophozoites, compared to only 30% of ovariectomizeed and placebo treated female mice and 10% of uncastrated and placebo treated female mice (*p*<0.02, *p*<0.0002,) (Fig. 2D). In contrast, parallel experiments of estradiol substitution in naive or orchiectomized male mice had no effect on abscess sizes and parasite survival rates compared to respective placebo controls (data not shown).

### Gender Differences in IFNγ Production of αGalCer or EhLPPG Activated Murine NKT Cells

**Figure 3:**
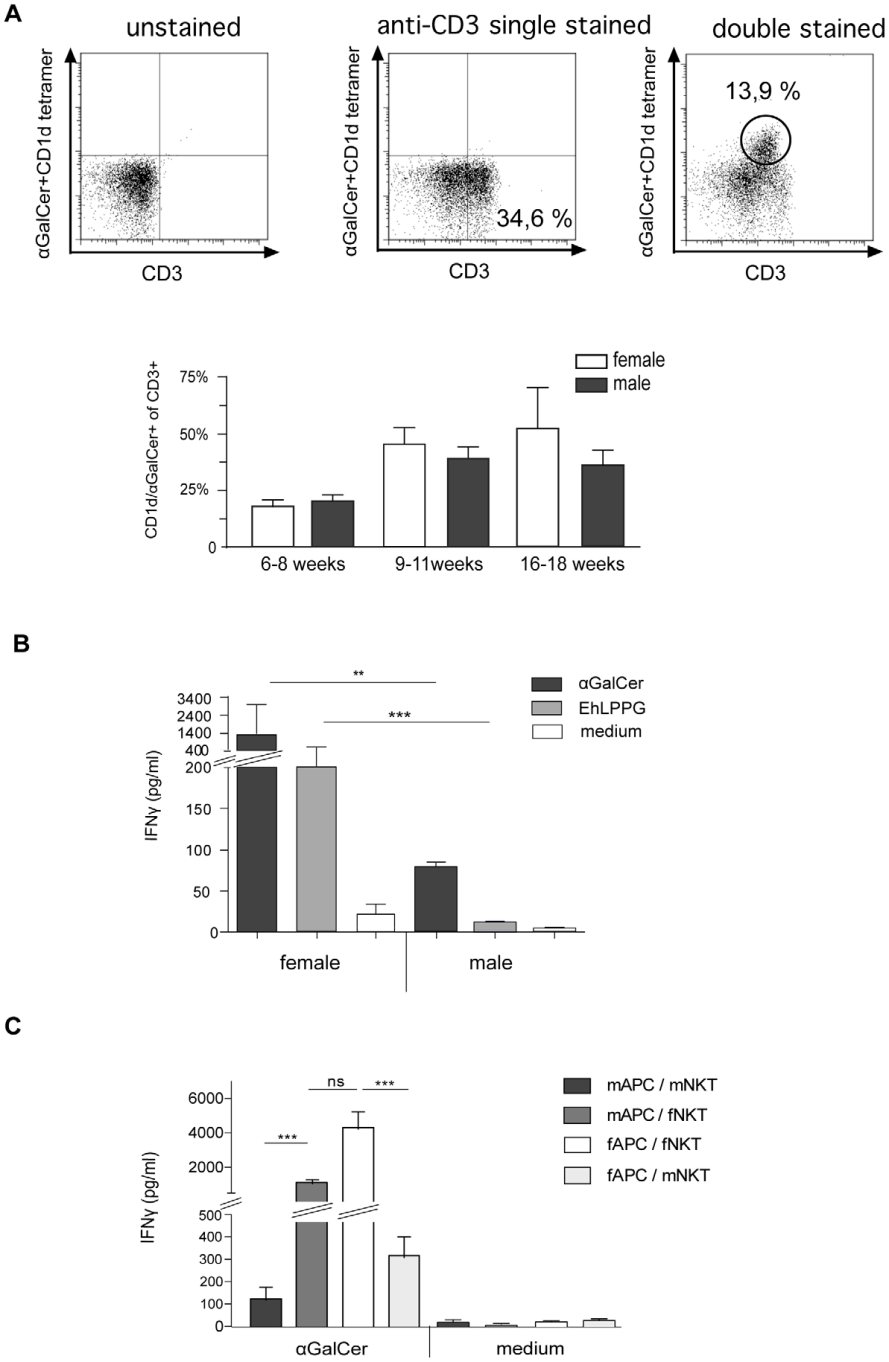
Characterization of NKT cells frequencies and NKT cell specific IFNγ production in female and male mice. A) NKT cell frequencies in the liver of female and male mice at different ages are shown. NKT cell numbers were determined as percentage of αGalCer -CD1d tetramer positive cells to CD3^+^ T cells. B) IFN**γ** production of NKT cells sorted (αGalCer -CD1d tetramer) from the liver of female or male mice after 48 h of co-incubation with αGalCer (4 µg/ml) or EhLLPG (8 µg/ml) pre-pulsed APCs. Medium control includes NKT cells co-cultured with naive APC’s. IFN**γ** production was quantified by ELISA (5 independent experiments were performed, a summary of data is expressed as mean +/− SD, statistics: student t test). C) IFN**γ** production of male or female NKT cells upon stimulation with αGalCer pre-pulsed male or female APCs.

We have recently shown that NKT cells are critically important for the control of ALA and that an *E. histolytica* lipopeptidophosphoglycan (EhLPPG), similar to αGalCer, specifically activates liver or spleen murine NKT cells to secrete significant amounts of protective IFN**γ** [27]. To investigate whether NKT cells are responsible for gender-related differences, the frequencies of αGalCer -CD1d tetramer/CD3+ positive cells in the liver of female and male mice at different ages were determined (Fig. 3A). The results indicated lower numbers of NKT cells in young mice (6–8 weeks) compared to older animals (9–11 or 16–18 weeks) but there were no statistically significant differences between the genders. The activation of isolated NKT cells by αGalCer or EhLPPG pre-pulsed APCs generated from untreated animals induced significant higher amounts of IFN**γ** in female compared to male derived cells (αGalCer, *p*<0.003; EhLPPG, *p*<0.0001) (Fig. 3B). To further determine whether this gender specific IFN**γ** secretion is indeed due to NKT cells and not a result of a gender specific presentation by the corresponding APCs, activation of male or female NKT cells by αGalCer pre-pulsed APCs were performed using APCs either of the homologous or of the heterologous gender (Fig. 3C). The results clearly indicate that higher secretion of IFN**γ** in female mice is primarily a function of NKT cells, as it was independent from the gender of the APCs.

### Mouse CD4− NKT Cells Rather than CD4+ NKT Cells are Responsible for Gender Specific Differences in IFNγ Production upon Activation

**Figure 4:**
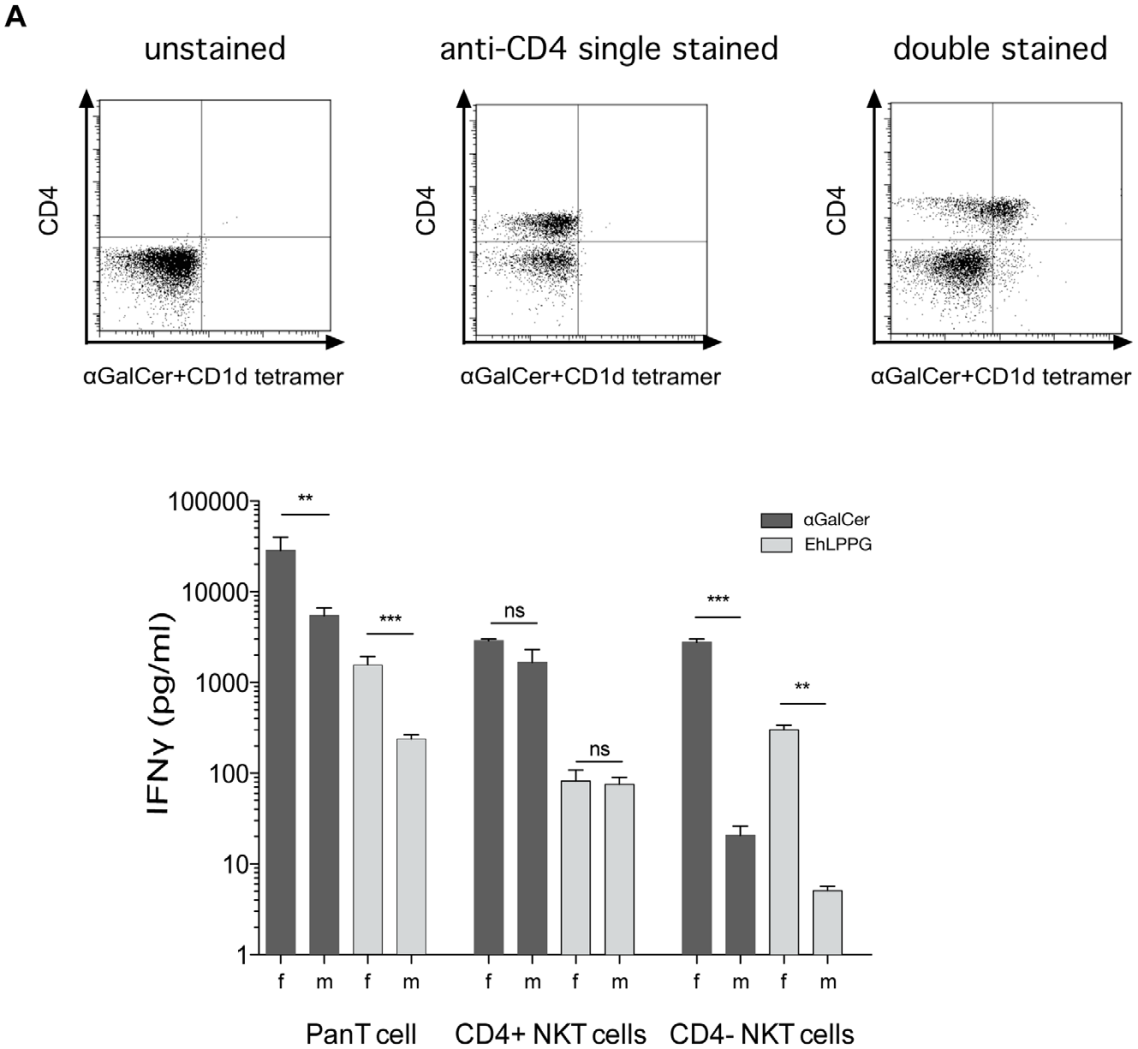
Characterization of the IFNγ producing gender specific murine NKT cell subpopulation. NKT cell subsets (PE- αGalCer -CD1d tetramer/CD4^+^) from female and male mice. IFN**γ** was determined following 48 h of co-cultivation with pre-pulsed APCs as described above (2 independent experiments were performed, a summary of data is expressed as mean +/− SD, statistics: student t test).

To further determine the NKT cell subpopulation that might be responsible for the observed gender specific differences, isolated NKT cells were further subdivided by the presence or absence of the surface marker CD4. NKT cell stimulation assays with αGalCer or EhLPPG indicate that both CD4+ and CD4− NKT cells secrete IFN**γ**. However, whereas we found no gender specific differences in the number of CD4− NKT cells (data not shown), CD4− NKT cells from female mice produced significant higher amounts of IFN**γ** (Fig. 4).

### Testosterone Reduces IFNγ Production of Activated Murine NKT Cells

**Figure 5:**
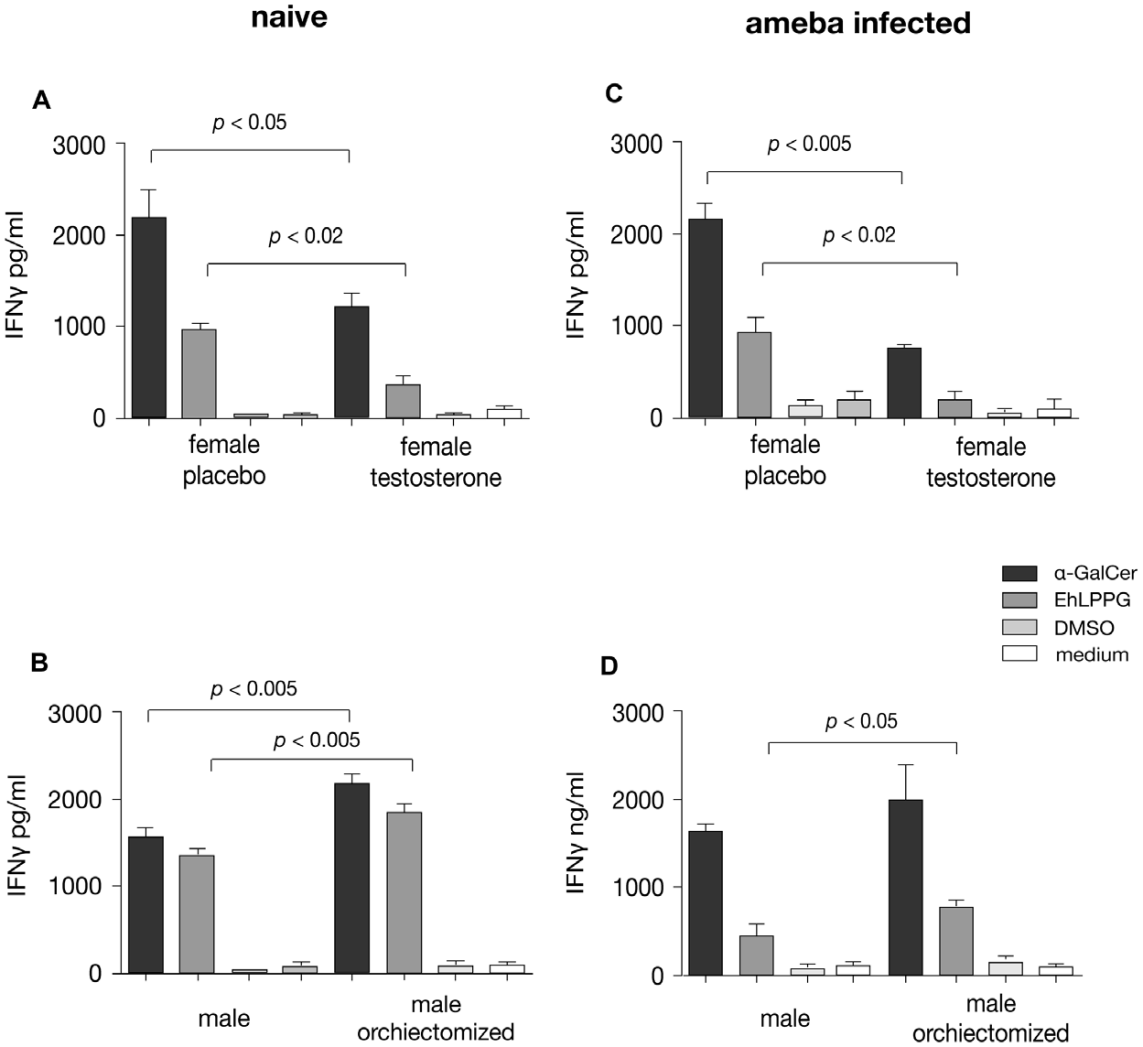
Testosterone modulates murine NKT cell specific IFNγ production. A) Liver lymphocytes from placebo or testosterone treated naïve female mice were gradient purified, T cells were isolated by magnetic cell sorting and co-cultured for 48 h with αGalCer (2 µg/ml) or EhLPPG (4 µg/ml) pre-pulsed APCs. Medium control comprises NKT cells co-cultured with naive APC’s. IFN**γ** in the supernatant was quantified using ELISA. B) Liver lymphocytes were isolated from naïve male or orchiectomized male mice and co-cultured with pre-pulsed APCs as described above. IFN**γ** production was quantified using ELISA. C/D) Identical experiments as performed in A and B but cells were isolated from the livers of ameba-infected female and male mice- Three independent experiments were performed (data express mean +/− SD, statistics: student`s t test).

To determine whether gender specific differences in IFN**γ** secretion of NKT cells following stimulation with EhLPPG is influenced by testosterone, purified liver lymphocytes from female and testosterone substituted female mice as well as from male and orchiectomized male mice were prepared. NKT cells present in these preparations were specifically activated upon co-culture with αGalCer - or EhLPPG-pulsed APCs (Fig. 5). Experiments were performed with cells from naïve (Fig. 5A u. B) as well as from ameba infected mice (Fig. 5C u. D). Analysis of IFN**γ** in supernatants of induced cells clearly indicated a testosterone dependent inhibitory effect on IFN**γ** secretion by NKT cells as αGalCer - or EhLPPG-induced IFN**γ** production was significant lower in liver derived NKT cells from testosterone treated animals compared to respective female controls (αGalCer, *p_naive_*<0.05, *p*_infected_<0.005; EhLPPG, *p_naive_*<0.02, *p*_infected_<0.02) (Fig. 5A u. C). In addition, deprivation of testosterone in male mice by orchiectomy significantly increased αGalCer - or EhLPPG-stimulated IFN**γ** production (EhLPPG, *p_naive_*<0.005, *p*_infected_<0.05) (Fig. 5B u. D).

## Discussion

To further elucidate the mechanisms responsible for the higher susceptibility of males to develop ALA we have used a mouse model for the disease to investigate the role of sexual hormones and in particular the contribution of testosterone and of NKT cells. The mouse model used in our study is considered suitable for the analysis of ALA associated immune reactions and gender related functions as the histopathological cellular infiltrates of parasite infected livers and the strong gender bias towards males indicates clear similarities to findings in human ALA patients [20].

Because of the complex and strong host specificity of human parasites, rodent models that enable studies on the influence of hormones on infectious diseases are rare and often do not reflect the same gender bias that occurs in humans [31]. However, in rodent models for leishmaniasis, a parasitic disease with a male preference in humans [1], [15], male mice bear higher parasite burden in the liver compared to female mice [15]. In addition, androgen deprivation by orchiectomy decreases the number of leishmania amastigotes in the liver while testosterone treatment leads to the opposite effect [10]. Although the administration of testosterone substitution varies widely, comparable results are reported from other mouse models of infectious diseases such as malaria [11], [32], [33] [34], strongyloidiasis [35], schistosomiasis [36], trypanosomiasis [37] or tuberculosis [38].

There are numerous examples of infectious diseases with predispositions in males. In general, this phenomenon is attributed to a reduced ability of male individuals to generate an effective humoral or cellular immune response [1], [2], [39], [40]. However, the extent of gender differences in most infectious diseases is usually low. In the case of ALA, gender differences are substantial with a male to female ratio of up to 7∶1 in adult individuals depending on age [4]. Previous findings that the risk for ALA (i) is low and gender independent in children, (ii) increases after puberty in males, and (iii) shows peak incidence in middle-age men suggests that sexual hormones, in particular testosterone, might play a role in the susceptibility to ALA. The results presented here, strongly support this assumption. Gonadectomy selectively reduced the sizes of abscesses in male mice but did not alter susceptibility to ALA in female mice. On the other hand, substitution of testosterone in both ovariectomized female mice and untreated female mice significantly increased susceptibility to ALA as reflected by increases in abscess sizes and parasite survival rates in infected livers. In contrast, substitution of estradiol in male mice had no effect on ALA formation.

Previous studies have shown that NKT cells and IFN**γ** are critically important for the control of ALA in a mouse model for the disease and that NKT cells can be specifically activated not only by αGalCer but similarly by EhLPPG to produce protective IFN**γ** [20], [27]. Accordingly, to assess possible gender dependent immune functions underlying the testosterone dependent decrease in the control of ALA we have focused on the activation of murine NKT cells and their production of IFN**γ**. Our results clearly indicate increased IFN**γ** production by αGalCer or EhLPPG stimulated NKT cells from female mice compared to male mice, which is in line with earlier studies, indicating higher amounts of IFN**γ** in the serum of female mice after administration of αGalCer [41]. Differences in the amount of IFN**γ** production between male and female mice are obviously not the result of differences in the numbers of NKT cells, as in our *in vitro* experiments identical numbers of isolated NKT cells were used. Furthermore the quantification of liver-derived NKT cells from mice at various ages did not reveal significant differences between the genders.

Interestingly, cytokine production of murine NKT cells can be modulated by testosterone. Liver lymphocytes isolated from testosterone substituted female mice produced significantly less IFN**γ** upon stimulation with αGalCer or EhLPPG compared to female mice, while orchiectomy increased NKT cell dependent IFN**γ** secretion in male mice. These results were independent whether cells were isolated from naïve or from ameba infected mice. Testosterone, or steroid hormones in general, have been shown to activate lymphocyte immune functions either directly or via the modulation of macrophages [2], [42], [43], [44], [45]. In the mouse model, however, it is likely that testosterone affects NKT cells directly rather then via modulation of APC functions since the results were independent whether the APCs used for stimulation were prepared from gender matched or unmatched control mice. Our results indicate that the increased IFN**γ** production in response to stimulation with EhLPPG in female mice is not due to an expansion of NKT cells but primarily due to IFN**γ** secretion by CD4− NKT cells, which are also present in male mice but without producing significant amounts of IFN**γ**. However, it remains to be determined whether NKT cells possess plasma membrane, intracellular or nuclear androgen receptors as conventional T lymphocytes do [46], [47], [48]. Moreover, studies are required to establish the importance of our findings in mice for the observed gender differences in human amoebiasis. It has to be considered that compared to humans, mice contain substantially more NKT cells, in particular within the liver [49]and that there are differences in NKT cell subsets between mice and men. Previous studies on the frequency of peripheral blood NKT cells in healthy blood donors [50], [51], [52] or cancer patients [53] indicated gender-related differences with higher NKT cell numbers in females. On the contrary, in one recent study this finding could not be confirmed [54]. Human NKT cell subsets consist of CD4^+^, CD4^−^CD8^+^(DN) and CD8^+^ NKT cells. In contrast, mice lack CD8 as a marker for NKT cells. Thus, murine NKT cells are subdivided by the presence or absence of the CD4 marker only [23], [55]. In agreement with our observations in mice, the CD4^−^CD8^−^ NKT cell subset in humans exhibits a Th1-biased cytokine profile (IFN**γ**, MIP-1α and TNFα) [55], [56], [57] while the CD4^+^CD8^+^ NKT cell subset in human is supposed to induce a Th2-type immune response similar to the CD4+NKT cell subset in mice [55], [58]. Although gender differences in the circulating human NKT cell subsets have not been observed, a comparison of the functional NKT cell profiles between males and females revealed higher frequencies of cells producing IFN**γ** and MIP1-α in males but similar frequencies of cells in both genders producing IL-4 [54]. However, although gender distribution and functional activity of NKT cells might be similar among mice and human, differences in frequencies and organ distribution occur. In general, NKT cell numbers in humans are lower compared to mice. NKT cell frequencies among peripheral blood lymphocytes range between 0.01–1.0% in humans and between 0.2–1,0% in mice. The differences are more pronounced in the liver, where human NKT cells represent not more than 1% of the lymphocyte population while in mice 20–40% of liver lymphocytes are NKT cells [49], [59].

## References

[1] Roberts CW, Walker W, Alexander J (2001) Sex-associated hormones and immunity to protozoan parasites. Clin Microbiol Rev 14: 476–488. 11432809 10.1128/CMR.14.3.476-488.2001 PMC88985

[2] Klein SL (2004) Hormonal and immunological mechanisms mediating sex differences in parasite infection. Parasite Immunol 26: 247–264. 15541029 10.1111/j.0141-9838.2004.00710.x

[3] Acuna-Soto R, Maguire JH, Wirth DF (2000) Gender distribution in asymptomatic and invasive amebiasis. Am J Gastroenterol 95: 1277–1283. 10811339 10.1111/j.1572-0241.2000.01525.x

[4] Blessmann J, Van Linh P, Nu PA, Thi HD, Muller-Myhsok B, et al (2002) Epidemiology of amebiasis in a region of high incidence of amebic liver abscess in central Vietnam. Am J Trop Med Hyg 66: 578–583. 12201594 10.4269/ajtmh.2002.66.578

[5] Blessmann J, Ali IK, Nu PA, Dinh BT, Viet TQ, et al (2003) Longitudinal study of intestinal Entamoeba histolytica infections in asymptomatic adult carriers. J Clin Microbiol 41: 4745–4750. 14532214 10.1128/JCM.41.10.4745-4750.2003 PMC294961

[6] Barnes PF, De Cock KM, Reynolds TN, Ralls PW (1987) A comparison of amebic and pyogenic abscess of the liver. Medicine (Baltimore) 66: 472–483. 3316923 10.1097/00005792-198711000-00005

[7] Walderich B, Weber A, Knobloch J (1997) Differentiation of Entamoeba histolytica and Entamoeba dispar from German travelers and residents of endemic areas. Am J Trop Med Hyg 57: 70–74. 9242322 10.4269/ajtmh.1997.57.70

[8] Shandera WX, Bollam P, Hashmey RH, Athey PA, Greenberg SB, et al (1998) Hepatic amebiasis among patients in a public teaching hospital. South Med J 91: 829–837. 9743053 10.1097/00007611-199809000-00005

[9] Travison TG, Araujo AB, O’Donnell AB, Kupelian V, McKinlay JB (2007) A population-level decline in serum testosterone levels in American men. J Clin Endocrinol Metab 92: 196–202. 17062768 10.1210/jc.2006-1375

[10] Mock BA, Nacy CA (1988) Hormonal modulation of sex differences in resistance to Leishmania major systemic infections. Infect Immun 56: 3316–3319. 3182082 10.1128/iai.56.12.3316-3319.1988 PMC259743

[11] Mossmann H, Benten WP, Galanos C, Freudenberg M, Kuhn-Velten WN, et al (1997) Dietary testosterone suppresses protective responsiveness to Plasmodium chabaudi malaria. Life Sci 60: 839–848. 9076323 10.1016/s0024-3205(97)00012-x

[12] Friedl R, Brunner M, Moeslinger T, Spieckermann PG (2000) Testosterone inhibits expression of inducible nitric oxide synthase in murine macrophages. Life Sci 68: 417–429. 11205891 10.1016/s0024-3205(00)00953-x

[13] Bebo BF Jr, Schuster JC, Vandenbark AA, Offner H (1999) Androgens alter the cytokine profile and reduce encephalitogenicity of myelin-reactive T cells. J Immunol 162: 35–40. 9886367

[14] Zhang H, Zhao J, Wang P, Qiao Z (2001) Effect of testosterone on Leishmania donovani infection of macrophages. Parasitol Res 87: 674–676. 11511007 10.1007/s004360000354

[15] Travi BL, Arteaga LT, Leon AP, Adler GH (2002) Susceptibility of spiny rats (Proechimys semispinosus) to Leishmania (Viannia) panamensis and Leishmania (Leishmania) chagasi. Mem Inst Oswaldo Cruz 97: 887–892. 12386716 10.1590/s0074-02762002000600025

[16] Liu L, Wang L, Zhao Y, Wang Y, Wang Z, et al (2006) Testosterone attenuates p38 MAPK pathway during Leishmania donovani infection of macrophages. Parasitol Res 99: 189–193. 16547729 10.1007/s00436-006-0168-1

[17] Salata RA, Murray HW, Rubin BY, Ravdin JI (1987) The role of gamma interferon in the generation of human macrophages cytotoxic for Entamoeba histolytica trophozoites. Am J Trop Med Hyg 37: 72–78. 2886071 10.4269/ajtmh.1987.37.72

[18] Denis M, Chadee K (1989) Cytokine activation of murine macrophages for in vitro killing of Entamoeba histolytica trophozoites. Infect Immun 57: 1750–1756. 2542164 10.1128/iai.57.6.1750-1756.1989 PMC313351

[19] Seydel KB, Smith SJ, Stanley SL Jr (2000) Innate immunity to amebic liver abscess is dependent on gamma interferon and nitric oxide in a murine model of disease. Infect Immun 68: 400–402. 10603416 10.1128/iai.68.1.400-402.2000 PMC97149

[20] Lotter H, Jacobs T, Gaworski I, Tannich E (2006) Sexual dimorphism in the control of amebic liver abscess in a mouse model of disease. Infect Immun 74: 118–124. 16368964 10.1128/IAI.74.1.118-124.2006 PMC1346632

[21] Bendelac A, Lantz O, Quimby ME, Yewdell JW, Bennink JR, et al (1995) CD1 recognition by mouse NK1+ T lymphocytes. Science 268: 863–865. 7538697 10.1126/science.7538697

[22] Porcelli SA, Modlin RL (1999) The CD1 system: antigen-presenting molecules for T cell recognition of lipids and glycolipids. Annu Rev Immunol 17: 297–329. 10358761 10.1146/annurev.immunol.17.1.297

[23] Godfrey DI, MacDonald HR, Kronenberg M, Smyth MJ, Van Kaer L (2004) NKT cells: what’s in a name? Nat Rev Immunol 4: 231–237. 15039760 10.1038/nri1309

[24] Tupin E, Kinjo Y, Kronenberg M (2007) The unique role of natural killer T cells in the response to microorganisms. Nat Rev Microbiol 5: 405–417. 17487145 10.1038/nrmicro1657

[25] Doisne JM, Soulard V, Becourt C, Amniai L, Henrot P, et al (2011) Cutting edge: crucial role of IL-1 and IL-23 in the innate IL-17 response of peripheral lymph node NK1.1- invariant NKT cells to bacteria. J Immunol 186: 662–666. 21169541 10.4049/jimmunol.1002725

[26] Wingender G, Krebs P, Beutler B, Kronenberg M (2010) Antigen-specific cytotoxicity by invariant NKT cells in vivo is CD95/CD178-dependent and is correlated with antigenic potency. J Immunol 185: 2721–2729. 20660713 10.4049/jimmunol.1001018 PMC2989418

[27] Lotter H, Gonzalez-Roldan N, Lindner B, Winau F, Isibasi A, et al (2009) Natural killer T cells activated by a lipopeptidophosphoglycan from Entamoeba histolytica are critically important to control amebic liver abscess. PLoS Pathog 5: e1000434. 19436711 10.1371/journal.ppat.1000434 PMC2674934

[28] Westphal O, Jann K, Himmelspach K (1983) Chemistry and immunochemistry of bacterial lipopolysaccharides as cell wall antigens and endotoxins. Prog Allergy 33: 9–39. 6187018

[29] Lutz MB, Kukutsch N, Ogilvie AL, Rossner S, Koch F, et al (1999) An advanced culture method for generating large quantities of highly pure dendritic cells from mouse bone marrow. J Immunol Methods 223: 77–92. 10037236 10.1016/s0022-1759(98)00204-x

[30] Volkmann A, Neefjes J, Stockinger B (1996) A conditionally immortalized dendritic cell line which differentiates in contact with T cells or T cell-derived cytokines. European journal of immunology 26: 2565–2572. 8921940 10.1002/eji.1830261105

[31] Morales-Montor J, Chavarria A, De Leon MA, Del Castillo LI, Escobedo EG, et al (2004) Host gender in parasitic infections of mammals: an evaluation of the female host supremacy paradigm. J Parasitol 90: 531–546. 15270097 10.1645/GE-113R3

[32] Benten WP, Ulrich P, Kuhn-Velten WN, Vohr HW, Wunderlich F (1997) Testosterone-induced susceptibility to Plasmodium chabaudi malaria: persistence after withdrawal of testosterone. J Endocrinol 153: 275–281. 9166117 10.1677/joe.0.1530275

[33] Liu L, Benten WP, Wang L, Hao X, Li Q, et al (2005) Modulation of Leishmania donovani infection and cell viability by testosterone in bone marrow-derived macrophages: signaling via surface binding sites. Steroids 70: 604–614. 15964042 10.1016/j.steroids.2005.02.020

[34] Krucken J, Dkhil MA, Braun JV, Schroetel RM, El-Khadragy M, et al (2005) Testosterone suppresses protective responses of the liver to blood-stage malaria. Infect Immun 73: 436–443. 15618182 10.1128/IAI.73.1.436-443.2005 PMC538982

[35] Watanabe K, Hamano S, Noda K, Koga M, Tada I (1999) Strongyloides ratti: additive effect of testosterone implantation and carbon injection on the susceptibility of female mice. Parasitol Res 85: 522–526. 10382601 10.1007/s004360050591

[36] Nakazawa M, Fantappie MR, Freeman GL Jr, Eloi-Santos S, Olsen NJ, et al (1997) Schistosoma mansoni: susceptibility differences between male and female mice can be mediated by testosterone during early infection. Exp Parasitol 85: 233–240. 9085920 10.1006/expr.1997.4148

[37] Filipin Mdel V, Brazao V, Caetano LC, Santello FH, Toldo MP, et al (2008) Trypanosoma cruzi: orchiectomy and dehydroepiandrosterone therapy in infected rats. Exp Parasitol 120: 249–254. 18700143 10.1016/j.exppara.2008.07.012

[38] Yamamoto Y, Saito H, Setogawa T, Tomioka H (1991) Sex differences in host resistance to Mycobacterium marinum infection in mice. Infect Immun 59: 4089–4096. 1937768 10.1128/iai.59.11.4089-4096.1991 PMC259001

[39] Marriott I, Huet-Hudson YM (2006) Sexual dimorphism in innate immune responses to infectious organisms. Immunol Res 34: 177–192. 16891670 10.1385/IR:34:3:177

[40] Neyrolles O, Quintana-Murci L (2009) Sexual inequality in tuberculosis. PLoS Med 6: e1000199. 20027210 10.1371/journal.pmed.1000199 PMC2788129

[41] Gourdy P, Araujo LM, Zhu R, Garmy-Susini B, Diem S, et al (2005) Relevance of sexual dimorphism to regulatory T cells: estradiol promotes IFN-{gamma} production by invariant natural killer T cells. Blood 105: 2415–2420. 15383462 10.1182/blood-2004-07-2819

[42] Cutolo A, Isernia T, Izzo I, Pierri R, Zeni L (1995) Transverse mode analysis of a laser beam by near- and far-field intensity measurements. Appl Opt 34: 7974–7978. 21068894 10.1364/AO.34.007974

[43] Kanda N, Tsuchida T, Tamaki K (1996) Testosterone inhibits immunoglobulin production by human peripheral blood mononuclear cells. Clin Exp Immunol 106: 410–415. 8918592 10.1046/j.1365-2249.1996.d01-842.x PMC2200579

[44] Wunderlich F, Benten WP, Lieberherr M, Guo Z, Stamm O, et al (2002) Testosterone signaling in T cells and macrophages. Steroids 67: 535–538. 11960632 10.1016/s0039-128x(01)00175-1

[45] Bouman A, Heineman MJ, Faas MM (2005) Sex hormones and the immune response in humans. Hum Reprod Update 11: 411–423. 15817524 10.1093/humupd/dmi008

[46] Chandler VL, Maler BA, Yamamoto KR (1983) DNA sequences bound specifically by glucocorticoid receptor in vitro render a heterologous promoter hormone responsive in vivo. Cell 33: 489–499. 6190571 10.1016/0092-8674(83)90430-0

[47] Benten WP, Lieberherr M, Giese G, Wunderlich F (1998) Estradiol binding to cell surface raises cytosolic free calcium in T cells. FEBS Lett 422: 349–353. 9498814 10.1016/s0014-5793(98)00039-8

[48] Benten WP, Lieberherr M, Giese G, Wrehlke C, Stamm O, et al (1999) Functional testosterone receptors in plasma membranes of T cells. Faseb J 13: 123–133. 9872937 10.1096/fasebj.13.1.123

[49] Subleski JJ, Ortaldo JR (2009) Editorial: NKT cells: to suppress or not to suppress, that is the question. Journal of leukocyte biology 86: 751–752. 19797301 10.1189/jlb.0309118 PMC2752013

[50] Sandberg JK, Bhardwaj N, Nixon DF (2003) Dominant effector memory characteristics, capacity for dynamic adaptive expansion, and sex bias in the innate Valpha24 NKT cell compartment. European journal of immunology 33: 588–596. 12616479 10.1002/eji.200323707

[51] Montoya CJ, Pollard D, Martinson J, Kumari K, Wasserfall C, et al (2007) Characterization of human invariant natural killer T subsets in health and disease using a novel invariant natural killer T cell-clonotypic monoclonal antibody, 6B11. Immunology 122: 1–14. 17662044 10.1111/j.1365-2567.2007.02647.x PMC2265989

[52] Kee SJ, Park YW, Cho YN, Jin HM, Kim MJ, et al (2012) Age- and gender-related differences in circulating natural killer T cells and their subset levels in healthy Korean adults. Human immunology 73: 1011–1016. 22884979 10.1016/j.humimm.2012.07.335

[53] Molling JW, Kolgen W, van der Vliet HJ, Boomsma MF, Kruizenga H, et al (2005) Peripheral blood IFN-gamma-secreting Valpha24+Vbeta11+ NKT cell numbers are decreased in cancer patients independent of tumor type or tumor load. International journal of cancer Journal international du cancer 116: 87–93. 15756674 10.1002/ijc.20998

[54] Snyder-Cappione JE, Tincati C, Eccles-James IG, Cappione AJ, Ndhlovu LC, et al (2010) A comprehensive ex vivo functional analysis of human NKT cells reveals production of MIP1-alpha and MIP1-beta, a lack of IL-17, and a Th1-bias in males. PloS one 5: e15412. 21082024 10.1371/journal.pone.0015412 PMC2972714

[55] Godfrey DI, Stankovic S, Baxter AG (2010) Raising the NKT cell family. Nature immunology 11: 197–206. 20139988 10.1038/ni.1841

[56] Takahashi T, Chiba S, Nieda M, Azuma T, Ishihara S, et al (2002) Cutting edge: analysis of human V alpha 24+CD8+ NK T cells activated by alpha-galactosylceramide-pulsed monocyte-derived dendritic cells. Journal of immunology 168: 3140–3144. 10.4049/jimmunol.168.7.3140 11907064

[57] He Y, Xiao R, Ji X, Li L, Chen L, et al (2010) EBV promotes human CD8 NKT cell development. PLoS pathogens 6: e1000915. 20502687 10.1371/journal.ppat.1000915 PMC2873918

[58] Mannik LA, Chin-Yee I, Sharif S, Van Kaer L, Delovitch TL, et al (2011) Engagement of glycosylphosphatidylinositol-anchored proteins results in enhanced mouse and human invariant natural killer T cell responses. Immunology 132: 361–375. 21070234 10.1111/j.1365-2567.2010.03369.x PMC3044902

[59] Kenna T, Golden-Mason L, Porcelli SA, Koezuka Y, Hegarty JE, et al (2003) NKT cells from normal and tumor-bearing human livers are phenotypically and functionally distinct from murine NKT cells. Journal of immunology 171: 1775–1779. 10.4049/jimmunol.171.4.1775 12902477

